# Correction: Association of Polymorphism rs198977 in Human Kallikrein-2 Gene (KLK2) with Susceptibility of Prostate Cancer: A Meta-Analysis

**DOI:** 10.1371/annotation/9e4eacfb-5de5-44f5-b027-b694f35e370e

**Published:** 2013-08-26

**Authors:** Lishan Wang

The content of this Correction is superseded by the Retraction [2] and has been updated on June 7, 2022.
